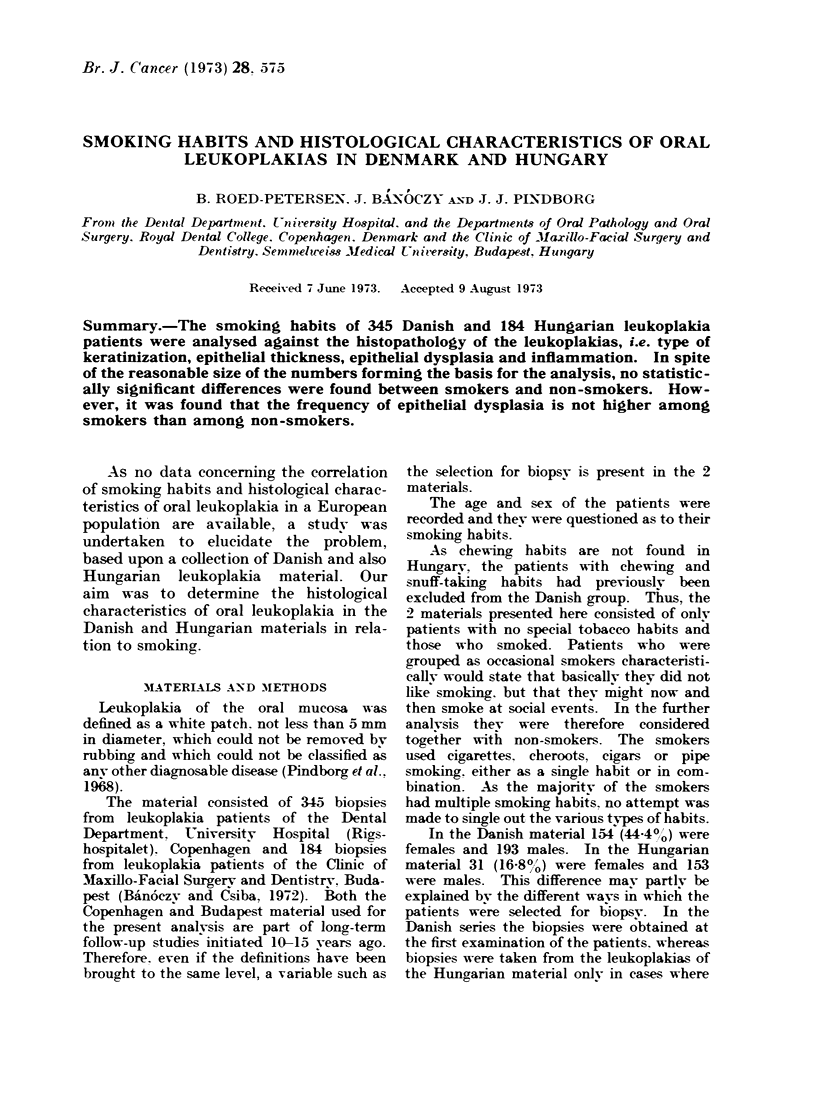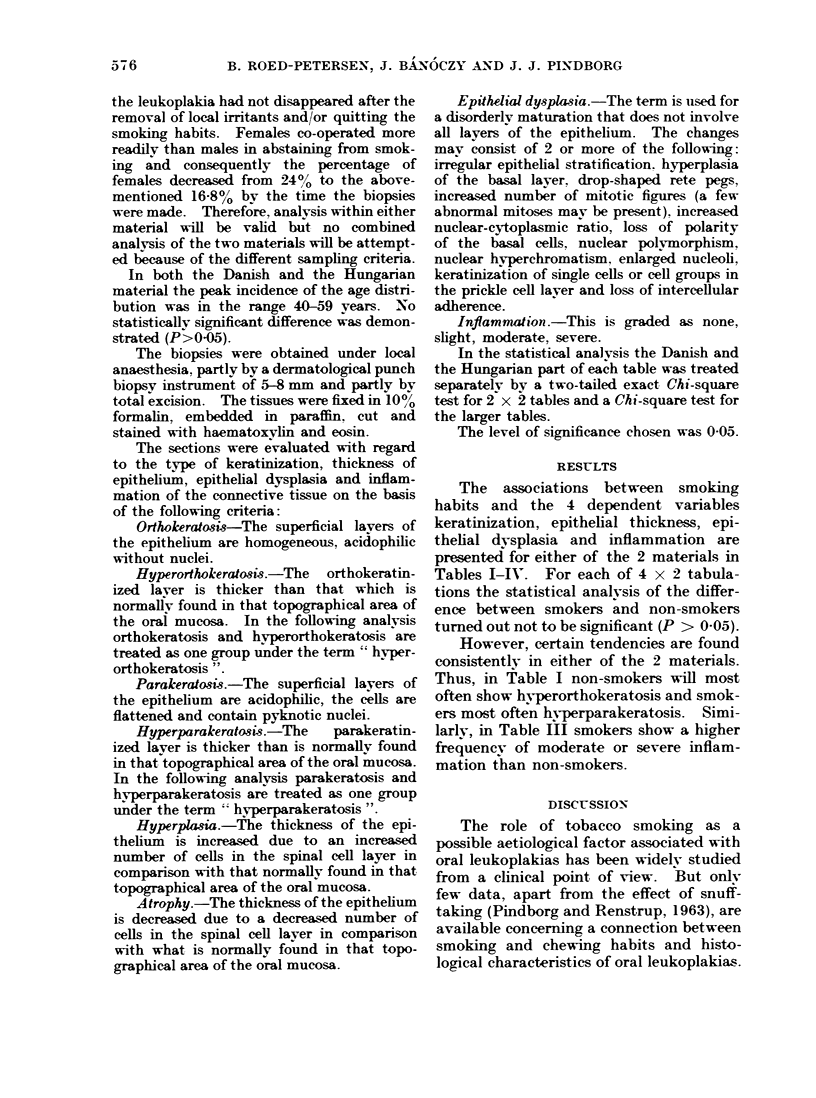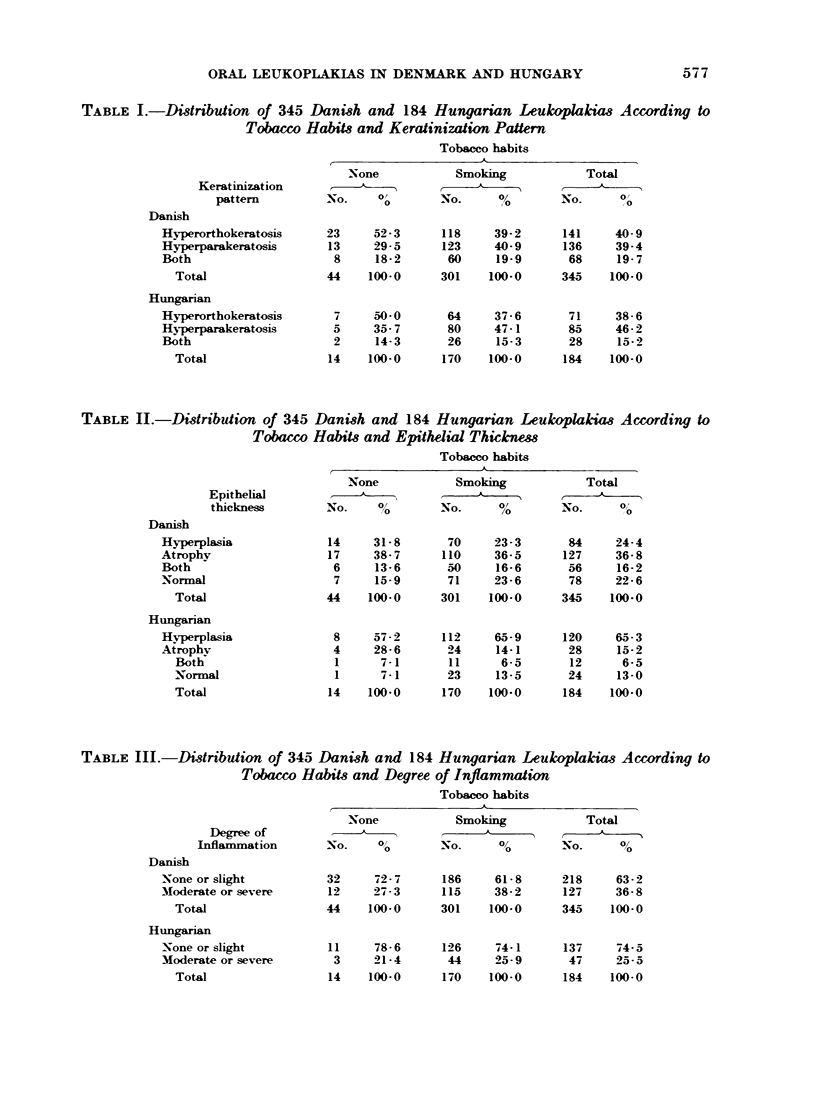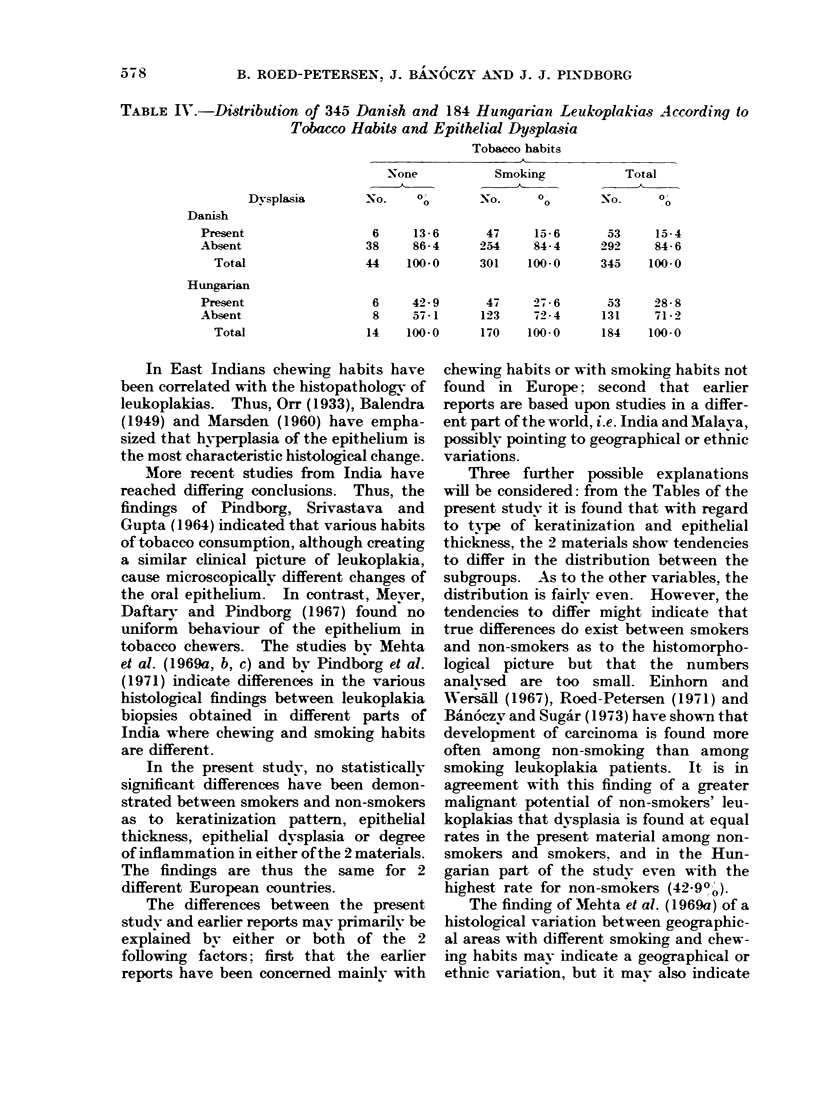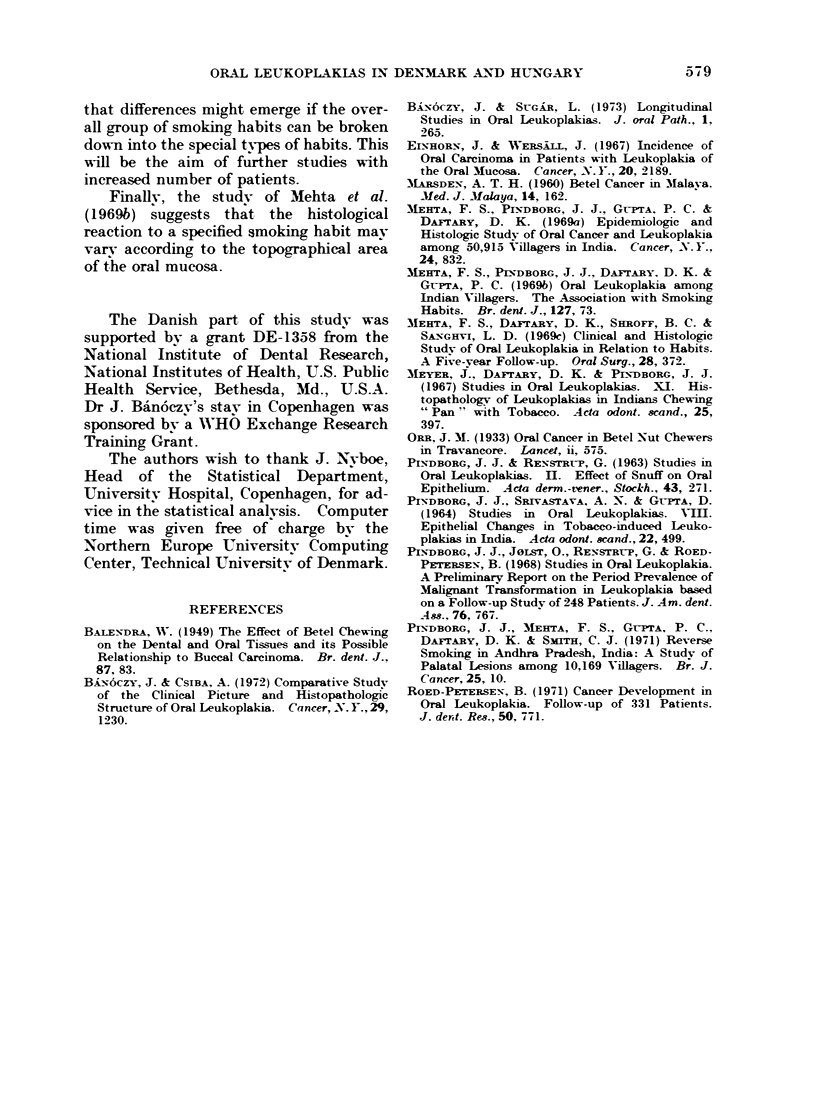# Smoking Habits and Histological Characteristics of Oral Leukoplakias in Denmark and Hungary

**DOI:** 10.1038/bjc.1973.188

**Published:** 1973-12

**Authors:** B. Roed-Petersen, J. Bánóczy, J. J. Pindborg

## Abstract

The smoking habits of 345 Danish and 184 Hungarian leukoplakia patients were analysed against the histopathology of the leukoplakias, *i.e.* type of keratinization, epithelial thickness, epithelial dysplasia and inflammation. In spite of the reasonable size of the numbers forming the basis for the analysis, no statistically significant differences were found between smokers and non-smokers. However, it was found that the frequency of epithelial dysplasia is not higher among smokers than among non-smokers.


*Br. J. Cancer* (1973) **28**, 575

### SMOKING HABITS AND HISTOLOGICAL CHARACTERISTICS OF ORAL LEUKOPLAKIAS IN DENMARK AND HUNGARY

B. ROED-PETERSEN, J. BÁNÓCZY AND J. J. PINDBORG

*From the Dental Department, University Hospital, and the Departments of Oral Pathology and Oral Surgery, Royal Dental College, Copenhagen, Denmark and the Clinic of Maxillo-Facial Surgery and Dentistry, Semmelweiss Medical University, Budapest, Hungary*

Received 7 June 1973. Accepted 9 August 1973

**Summary.—The smoking habits of 345 Danish and 184 Hungarian leukoplakia patients were analysed against the histopathology of the leukoplakias, *i.e.* type of keratinization, epithelial thickness, epithelial dysplasia and inflammation. In spite of the reasonable size of the numbers forming the basis for the analysis, no statistically significant differences were found between smokers and non-smokers. However, it was found that the frequency of epithelial dysplasia is not higher among smokers than among non-smokers.**

As no data concerning the correlation of smoking habits and histological characteristics of oral leukoplakia in a European population are available, a study was undertaken to elucidate the problem, based upon a collection of Danish and also Hungarian leukoplakia material. Our aim was to determine the histological characteristics of oral leukoplakia in the Danish and Hungarian materials in relation to smoking.

### MATERIALS AND METHODS

Leukoplakia of the oral mucosa was defined as a white patch, not less than 5 mm in diameter, which could not be removed by rubbing and which could not be classified as any other diagnosable disease (Pindborg *et al.*, 1968).

The material consisted of 345 biopsies from leukoplakia patients of the Dental Department, University Hospital (Rigshospitalet), Copenhagen and 184 biopsies from leukoplakia patients of the Clinic of Maxillo-Facial Surgery and Dentistry, Budapest (Bánóczy and Csiba, 1972). Both the Copenhagen and Budapest material used for the present analysis are part of long-term follow-up studies initiated 10–15 years ago. Therefore, even if the definitions have been brought to the same level, a variable such as the selection for biopsy is present in the 2 materials.

The age and sex of the patients were recorded and they were questioned as to their smoking habits.

As chewing habits are not found in Hungary, the patients with chewing and snuff-taking habits had previously been excluded from the Danish group. Thus, the 2 materials presented here consisted of only patients with no special tobacco habits and those who smoked. Patients who were grouped as occasional smokers characteristically would state that basically they did not like smoking, but that they might now and then smoke at social events. In the further analysis they were therefore considered together with non-smokers. The smokers used cigarettes, cheroots, cigars or pipe smoking, either as a single habit or in combination. As the majority of the smokers had multiple smoking habits, no attempt was made to single out the various types of habits.

In the Danish material 154 (44·4%) were females and 193 males. In the Hungarian material 31 (16·8%) were females and 153 were males. This difference may partly be explained by the different ways in which the patients were selected for biopsy. In the Danish series the biopsies were obtained at the first examination of the patients, whereas biopsies were taken from the leukoplakias of the Hungarian material only in cases where the leukoplakia had not disappeared after the removal of local irritants and/or quitting the smoking habits. Females co-operated more readily than males in abstaining from smoking and consequently the percentage of females decreased from 24% to the above-mentioned 16·8% by the time the biopsies were made. Therefore, analysis within either material will be valid but no combined analysis of the two materials will be attempted because of the different sampling criteria.

In both the Danish and the Hungarian material the peak incidence of the age distribution was in the range 40–59 years. No statistically significant difference was demonstrated ($P>0{\cdot}05$).

The biopsies were obtained under local anaesthesia, partly by a dermatological punch biopsy instrument of 5–8 mm and partly by total excision. The tissues were fixed in 10% formalin, embedded in paraffin, cut and stained with haematoxylin and eosin.

The sections were evaluated with regard to the type of keratinization, thickness of epithelium, epithelial dysplasia and inflammation of the connective tissue on the basis of the following criteria:

*Orthokeratosis*—The superficial layers of the epithelium are homogeneous, acidophilic without nuclei.

*Hyperorthokeratosis*.—The orthokeratinized layer is thicker than that which is normally found in that topographical area of the oral mucosa. In the following analysis orthokeratosis and hyperorthokeratosis are treated as one group under the term " hyperorthokeratosis ".

*Parakeratosis*.—The superficial layers of the epithelium are acidophilic, the cells are flattened and contain pyknotic nuclei.

*Hyperparakeratosis*.—The parakeratinized layer is thicker than is normally found in that topographical area of the oral mucosa. In the following analysis parakeratosis and hyperparakeratosis are treated as one group under the term " hyperparakeratosis ".

*Hyperplasia*.—The thickness of the epithelium is increased due to an increased number of cells in the spinal cell layer in comparison with that normally found in that topographical area of the oral mucosa.

*Atrophy*.—The thickness of the epithelium is decreased due to a decreased number of cells in the spinal cell layer in comparison with what is normally found in that topographical area of the oral mucosa.

*Epithelial dysplasia*.—The term is used for a disorderly maturation that does not involve all layers of the epithelium. The changes may consist of 2 or more of the following: irregular epithelial stratification, hyperplasia of the basal layer, drop-shaped rete pegs, increased number of mitotic figures (a few abnormal mitoses may be present), increased nuclear-cytoplasmic ratio, loss of polarity of the basal cells, nuclear polymorphism, nuclear hyperchromatism, enlarged nucleoli, keratinization of single cells or cell groups in the prickle cell layer and loss of intercellular adherence.

*Inflammation*.—This is graded as none, slight, moderate, severe.

In the statistical analysis the Danish and the Hungarian part of each table was treated separately by a two-tailed exact *Chi*-square test for 2 × 2 tables and a *Chi*-square test for the larger tables.

The level of significance chosen was 0·05.

### RESULTS

The associations between smoking habits and the 4 dependent variables keratinization, epithelial thickness, epithelial dysplasia and inflammation are presented for either of the 2 materials in Tables I–IV. For each of 4 × 2 tabulations the statistical analysis of the difference between smokers and non-smokers turned out not to be significant ($P > 0{\cdot}05$).

However, certain tendencies are found consistently in either of the 2 materials. Thus, in Table I non-smokers will most often show hyperorthokeratosis and smokers most often hyperparakeratosis. Similarly, in Table III smokers show a higher frequency of moderate or severe inflammation than non-smokers.

### DISCUSSION

The role of tobacco smoking as a possible aetiological factor associated with oral leukoplakias has been widely studied from a clinical point of view. But only few data, apart from the effect of snuff-taking (Pindborg and Renstrup, 1963), are available concerning a connection between smoking and chewing habits and histological characteristics of oral leukoplakias.

TABLE I.—*Distribution of* 345 *Danish and* 184 *Hungarian Leukoplakias According to Tobacco Habits and Keratinization Pattern*

| Keratinization pattern | Tobacco habits: None No. | % | Smoking No. | % | Total No. | % |
|---|---|---|---|---|---|---|
| Danish | | | | | | |
| Hyperorthokeratosis | 23 | 52·3 | 118 | 39·2 | 141 | 40·9 |
| Hyperparakeratosis | 13 | 29·5 | 123 | 40·9 | 136 | 39·4 |
| Both | 8 | 18·2 | 60 | 19·9 | 68 | 19·7 |
| Total | 44 | 100·0 | 301 | 100·0 | 345 | 100·0 |
| Hungarian | | | | | | |
| Hyperorthokeratosis | 7 | 50·0 | 64 | 37·6 | 71 | 38·6 |
| Hyperparakeratosis | 5 | 35·7 | 80 | 47·1 | 85 | 46·2 |
| Both | 2 | 14·3 | 26 | 15·3 | 28 | 15·2 |
| Total | 14 | 100·0 | 170 | 100·0 | 184 | 100·0 |

TABLE II.—*Distribution of* 345 *Danish and* 184 *Hungarian Leukoplakias According to Tobacco Habits and Epithelial Thickness*

| Epithelial thickness | Tobacco habits: None No. | % | Smoking No. | % | Total No. | % |
|---|---|---|---|---|---|---|
| Danish | | | | | | |
| Hyperplasia | 14 | 31·8 | 70 | 23·3 | 84 | 24·4 |
| Atrophy | 17 | 38·7 | 110 | 36·5 | 127 | 36·8 |
| Both | 6 | 13·6 | 50 | 16·6 | 56 | 16·2 |
| Normal | 7 | 15·9 | 71 | 23·6 | 78 | 22·6 |
| Total | 44 | 100·0 | 301 | 100·0 | 345 | 100·0 |
| Hungarian | | | | | | |
| Hyperplasia | 8 | 57·2 | 112 | 65·9 | 120 | 65·3 |
| Atrophy | 4 | 28·6 | 24 | 14·1 | 28 | 15·2 |
| Both | 1 | 7·1 | 11 | 6·5 | 12 | 6·5 |
| Normal | 1 | 7·1 | 23 | 13·5 | 24 | 13·0 |
| Total | 14 | 100·0 | 170 | 100·0 | 184 | 100·0 |

TABLE III.—*Distribution of* 345 *Danish and* 184 *Hungarian Leukoplakias According to Tobacco Habits and Degree of Inflammation*

| Degree of Inflammation | Tobacco habits: None No. | % | Smoking No. | % | Total No. | % |
|---|---|---|---|---|---|---|
| Danish | | | | | | |
| None or slight | 32 | 72·7 | 186 | 61·8 | 218 | 63·2 |
| Moderate or severe | 12 | 27·3 | 115 | 38·2 | 127 | 36·8 |
| Total | 44 | 100·0 | 301 | 100·0 | 345 | 100·0 |
| Hungarian | | | | | | |
| None or slight | 11 | 78·6 | 126 | 74·1 | 137 | 74·5 |
| Moderate or severe | 3 | 21·4 | 44 | 25·9 | 47 | 25·5 |
| Total | 14 | 100·0 | 170 | 100·0 | 184 | 100·0 |

TABLE IV.—*Distribution of* 345 *Danish and* 184 *Hungarian Leukoplakias According to Tobacco Habits and Epithelial Dysplasia*

| | Tobacco habits | | | | | |
|---|---|---|---|---|---|---|
| | None | | Smoking | | Total | |
| Dysplasia | No. | % | No. | % | No. | % |
| Danish | | | | | | |
| Present | 6 | 13·6 | 47 | 15·6 | 53 | 15·4 |
| Absent | 38 | 86·4 | 254 | 84·4 | 292 | 84·6 |
| Total | 44 | 100·0 | 301 | 100·0 | 345 | 100·0 |
| Hungarian | | | | | | |
| Present | 6 | 42·9 | 47 | 27·6 | 53 | 28·8 |
| Absent | 8 | 57·1 | 123 | 72·4 | 131 | 71·2 |
| Total | 14 | 100·0 | 170 | 100·0 | 184 | 100·0 |

In East Indians chewing habits have been correlated with the histopathology of leukoplakias. Thus, Orr (1933), Balendra (1949) and Marsden (1960) have emphasized that hyperplasia of the epithelium is the most characteristic histological change.

More recent studies from India have reached differing conclusions. Thus, the findings of Pindborg, Srivastava and Gupta (1964) indicated that various habits of tobacco consumption, although creating a similar clinical picture of leukoplakia, cause microscopically different changes of the oral epithelium. In contrast, Meyer, Daftary and Pindborg (1967) found no uniform behaviour of the epithelium in tobacco chewers. The studies by Mehta *et al.* (1969*a*, *b*, *c*) and by Pindborg *et al.* (1971) indicate differences in the various histological findings between leukoplakia biopsies obtained in different parts of India where chewing and smoking habits are different.

In the present study, no statistically significant differences have been demonstrated between smokers and non-smokers as to keratinization pattern, epithelial thickness, epithelial dysplasia or degree of inflammation in either of the 2 materials. The findings are thus the same for 2 different European countries.

The differences between the present study and earlier reports may primarily be explained by either or both of the 2 following factors; first that the earlier reports have been concerned mainly with chewing habits or with smoking habits not found in Europe; second that earlier reports are based upon studies in a different part of the world, *i.e.* India and Malaya, possibly pointing to geographical or ethnic variations.

Three further possible explanations will be considered: from the Tables of the present study it is found that with regard to type of keratinization and epithelial thickness, the 2 materials show tendencies to differ in the distribution between the subgroups. As to the other variables, the distribution is fairly even. However, the tendencies to differ might indicate that true differences do exist between smokers and non-smokers as to the histomorphological picture but that the numbers analysed are too small. Einhorn and Wersäll (1967), Roed-Petersen (1971) and Bánóczy and Sugár (1973) have shown that development of carcinoma is found more often among non-smoking than among smoking leukoplakia patients. It is in agreement with this finding of a greater malignant potential of non-smokers' leukoplakias that dysplasia is found at equal rates in the present material among non-smokers and smokers, and in the Hungarian part of the study even with the highest rate for non-smokers (42·9%).

The finding of Mehta *et al.* (1969*a*) of a histological variation between geographical areas with different smoking and chewing habits may indicate a geographical or ethnic variation, but it may also indicate that differences might emerge if the overall group of smoking habits can be broken down into the special types of habits. This will be the aim of further studies with increased number of patients.

Finally, the study of Mehta *et al.* (1969*b*) suggests that the histological reaction to a specified smoking habit may vary according to the topographical area of the oral mucosa.

The Danish part of this study was supported by a grant DE-1358 from the National Institute of Dental Research, National Institutes of Health, U.S. Public Health Service, Bethesda, Md., U.S.A. Dr J. Bánóczy's stay in Copenhagen was sponsored by a WHO Exchange Research Training Grant.

The authors wish to thank J. Nyboe, Head of the Statistical Department, University Hospital, Copenhagen, for advice in the statistical analysis. Computer time was given free of charge by the Northern Europe University Computing Center, Technical University of Denmark.

### REFERENCES

Balendra, W. (1949) The Effect of Betel Chewing on the Dental and Oral Tissues and its Possible Relationship to Buccal Carcinoma. *Br. dent. J.*, **87**, 83.

Bánóczy, J. & Csiba, A. (1972) Comparative Study of the Clinical Picture and Histopathologic Structure of Oral Leukoplakia. *Cancer, N.Y.*, **29**, 1230.

Bánóczy, J. & Sugár, L. (1973) Longitudinal Studies in Oral Leukoplakias. *J. oral Path.*, **1**, 265.

Einhorn, J. & Wersäll, J. (1967) Incidence of Oral Carcinoma in Patients with Leukoplakia of the Oral Mucosa. *Cancer, N.Y.*, **20**, 2189.

Marsden, A. T. H. (1960) Betel Cancer in Malaya. *Med. J. Malaya*, **14**, 162.

Mehta, F. S., Pindborg, J. J., Gupta, P. C. & Daftary, D. K. (1969*a*) Epidemiologic and Histologic Study of Oral Cancer and Leukoplakia among 50,915 Villagers in India. *Cancer, N.Y.*, **24**, 832.

Mehta, F. S., Pindborg, J. J., Daftary, D. K. & Gupta, P. C. (1969*b*) Oral Leukoplakia among Indian Villagers. The Association with Smoking Habits. *Br. dent. J.*, **127**, 73.

Mehta, F. S., Daftary, D. K., Shroff, B. C. & Sanghvi, L. D. (1969*c*) Clinical and Histologic Study of Oral Leukoplakia in Relation to Habits. A Five-year Follow-up. *Oral Surg.*, **28**, 372.

Meyer, J., Daftary, D. K. & Pindborg, J. J. (1967) Studies in Oral Leukoplakias. XI. Histopathology of Leukoplakias in Indians Chewing "Pan" with Tobacco. *Acta odont. scand.*, **25**, 397.

Orr, J. M. (1933) Oral Cancer in Betel Nut Chewers in Travancore. *Lancet*, ii, 575.

Pindborg, J. J. & Renstrup, G. (1963) Studies in Oral Leukoplakias. II. Effect of Snuff on Oral Epithelium. *Acta derm.-vener., Stockh.*, **43**, 271.

Pindborg, J. J., Srivastava, A. N. & Gupta, D. (1964) Studies in Oral Leukoplakias. VIII. Epithelial Changes in Tobacco-induced Leukoplakias in India. *Acta odont. scand.*, **22**, 499.

Pindborg, J. J., Jølst, O., Renstrup, G. & Roed-Petersen, B. (1968) Studies in Oral Leukoplakia. A Preliminary Report on the Period Prevalence of Malignant Transformation in Leukoplakia based on a Follow-up Study of 248 Patients. *J. Am. dent. Ass.*, **76**, 767.

Pindborg, J. J., Mehta, F. S., Gupta, P. C., Daftary, D. K. & Smith, C. J. (1971) Reverse Smoking in Andhra Pradesh, India: A Study of Palatal Lesions among 10,169 Villagers. *Br. J. Cancer*, **25**, 10.

Roed-Petersen, B. (1971) Cancer Development in Oral Leukoplakia. Follow-up of 331 Patients. *J. dent. Res.*, **50**, 771.